# *Rickettsia typhi* infection in severe fever with thrombocytopenia patients, China

**DOI:** 10.1080/22221751.2019.1599696

**Published:** 2019-04-08

**Authors:** Shao-Fei Zhang, Juan Du, Xian-Miao Mi, Qing-Bin Lu, Jie-Ying Bai, Ning Cui, Zhen-Dong Yang, Zhi-Bo Wang, Xiao-Ai Zhang, Pan-He Zhang, Hao Li, Wei Liu

**Affiliations:** a State Key Laboratory of Pathogen and Biosecurity, Beijing Institute of Microbiology and Epidemiology, Beijing Key Laboratory of Vector Borne and Natural Focus Infectious Diseases, Beijing, People’s Republic of China; b School of Public Health, Peking University, Beijing, People’s Republic of China; c Laboratory Animal Center, Academy of Military Medical Sciences, Beijing, People’s Republic of China; d The 154 Hospital, People's Liberation Army, Xinyang, People’s Republic of China

**Keywords:** *Rickettsia typhi*, severe fever with thrombocytopenia syndrome, vector-borne diseases, serological test, China

## Dear Editor,

Severe fever with thrombocytopenia (SFTS) is an emerging infectious disease caused by a novel bunyavirus (SFTSV) that was widely identified in mainland China [1]. Ticks have been recognized as the vectors involved in SFTSV transmission to humans, and several domestic animals are considered potential reservoir host [1]. Therefore people who live in rural areas or have intense contact with insects or animals are at a high-risk of acquiring SFTSV infection. The disease has also been reported in Korea and Japan, indicating the possibility of spreading in the wider geographic range [2,3]. Although infection with SFTSV is associated with a self-limiting illness in the majority of individuals, it can cause severe clinical disease in a proportion of individuals [4–6]. Some individuals with severe disease proceed to the critical phase, which is associated with multiorgan dysregulation, haemorrhagic manifestation, neurological symptoms; whereas those with self-limited disease proceed to the recovery phase without developing any of these complications [6]. But at the early phase of the disease, patients have only non-specific febrile illnesses, making it difficult to achieve clinical based diagnosis [4,5]. Furthermore, laboratory capacity to diagnose the causative etiological agents is often lacking in rural areas. Consequently, the determination of the etiological diversity that might be causing SFTS warrants investigation with intense efforts. In recent years, an increased number of vector-borne pathogens other than SFTSV has been detected from the clinically diagnosed SFTS patients, comprising species of spotted fever group rickettsiae (SFGR), Hantaan and Seoul viruses, *Orientia tsutsugamushi*, and *Anaplasma phagocytophilum* [7–10]. Moreover, the previous study found that only 27% of the SFTS like patients reported tick bite history [9], indicating that the patients might be either infested by other vectors or infected with other vector-borne pathogens. *Murine typhus* is a flea-borne febrile illness caused by *Rickettsia typhi*, and is hard to be diagnosed due to the non-specific clinical features [11]. In China mainland, *R. typhi* was only rarely reported to cause human infection. In a field epidemiological survey performed in Tianjin, the antibody positive rates determined by IFA for *R. typhi* ranged from 5.0% to 58.2% from 2007 to 2009, suggesting a high possibility of acquiring *R. typhi* infection in people living in rural areas in China [12]. In our sentinel hospital that recruited SFTS like patients in Xinyang city, although SFTSV and SFGR were found to be the most frequently seen tick-borne agents [9,10] the causative agents in a percentage of patients remained to be determined. On the other hand, *R. typhi* was indeed detected in the captured mice in the same region, with a positive rate of 6.45%, indicating the epidemic status of *R. typhi* in natural reservoir [13]. This has inspired us to undertake a clinically based surveillance study to explore the presence of *R. typhi* in SFTS endemic regions. The clinical symptoms and laboratory findings of patients infected with *R. typhi* were used to differentiate diagnosis with those of SFTSV infection.

This retrospective study was conducted in Xinyang city, Henan Province, where SFGR infection used to be determined from clinical diagnosed SFTS patients [9,10]. The region has a humid subtropical climate with annual precipitation of around 1100 millimetres. The natural landscape of the region is plain and mountain, and the main local agriculture activities include preparing land for cultivation, planting crops, pasturing cattle, and tea-picking activity, etc. From March to November, 2014–2015, patients with suspected vector-borne disease, which was defined as: fever with axillary temperature >38°C, and absence of a focal site of infection (e.g. pneumonia, urinary tract infection), and had a reported history of tick or other vector bite or animal contact, were recruited from the vector-borne disease clinic of the PLA 154 Hospital and the Shangcheng People’s Hospital. Paired blood samples were obtained from all consenting patients with the first and second blood collected at least more than two weeks apart. The molecular test of SFTSV, SFG rickettsiae, and other tick-borne agents, including *Anaplasma phygocytophilum*, *Babesia microti*, and *Borrelia burgdorferi sensu lato*, was performed in the acute serum samples by PCR or real-time PCR (Appendix Table A1), and those positive were excluded from the study. The patients with paired serum were tested for *R. typhi* specific IgG antibody by applying the indirect immunofluorescence assay (IFA) (Focus, USA). Titre of 1:64 of IgG antibody was considered as positive reaction. An acute infection of *R. typhi* was defined by seroconversion or a four-fold increase in titres of IgG antibodies between paired serum samples.

A total of 1348 hospitalized patients who had illnesses meeting the case definition were recruited in 2014–2015, among whom 767 patients were confirmed with single SFTSV infection, 73 were determined to be with SFGR infections, or infections with other tick-borne agents. The other 508 patients who were negative for SFTSV were subject to anti-*R. typhi* antibody test. Seroconversion was evident for 60 (11.8%) patients. The mean age of the patients was 58.4 ± 11.3 years, lower than that of the SFTSV infection (62.0 ± 12.3) (*P* = .028, Table 1). Totally 34 (56.7%) were female, comparable with SFTSV infection (450, 58.7%). All patients were farmers and reported recent contact with livestock animals such as cattle, sheep, goats, or dogs, whereas only nine patients additionally provided evidence of insect bites. The 60 patients of *R. typhi* infection sought medical care from April and August, and the majority of the patients (47, 78.3%) sought care between May-June, which aligned with the seasonal distribution of SFTS.

**Table1.** Demographic and clinical characteristics of the patients with *R. typhi* infection *vs*. SFTSV infection, 2014–2015, China.

Characteristics	*R. typhi* infection (*n *=* *60)	SFTS single infection (*n *=* *767)	*P*-value
**Demographic characteristics**			
Age, Mean ± SD, years	58.4±11.3	62.0±12.3	0.028*
Sex, female	34 (56.7)	450 (58.7)	0.762
Reported history of insect bite	9 (15.0)	91 (11.8)	0.473
Hospital duration, median (IQR)	10 (8–12)	7 (5–9)	<0.001*
Delay days from onset to admission, median (IQR)	5 (4–7)	5 (4–7)	0.039*
**Clinical manifestations**			
**Common signs**			
Fever	59 (98.3)	764 (99.6)	0.261
Fever duration, days, median (IQR)	5 (4–7)	5 (4–7)	0.039*
Maximum temperature, Mean±SD, °C	38.6±0.6	38.7±1.0	0.731
Chills	4 (6.7)	66 (8.6)	0.810
Malaise	56 (93.3)	741 (96.6)	0.267
Lymphadenectasis	32 (53.3)	338 (44.1)	0.165
Myalgia	42 (70.0)	625 (81.5)	0.030*
Dizziness	10 (16.7)	131 (17.1)	0.935
Headache	5 (8.3)	61 (8.0)	0.917
Rash	1 (1.7)	4 (0.5)	0.314
Arthralgia	1 (1.7)	10 (1.3)	0.566
**Respiratory syndrome**	**30 (50.0)**	**378 (49.3)**	**0.915**
Cough	30 (50.0)	373 (48.6)	0.838
Productive cough	24 (40.0)	279 (36.4)	0.575
**Gastrointestinal illness**	**54 (90.0)**	**659 (85.9)**	**0.377**
Anorexia	27 (45.0)	360 (46.9)	0.772
Nausea	41 (68.3)	496 (64.7)	0.567
Vomit	18 (30.0)	212 (27.6)	0.694
Abdominal pain	3 (5.0)	33 (4.3)	0.740
Abdominal distension	3 (5.0)	26 (3.4)	0.461
Diarrhoea	4 (6.7)	174 (22.7)	0.003*
**Hemorrhage manifestations**	**14 (23.3)**	**182 (23.7)**	**0.945**
Gingival bleeding	6 (10.0)	61 (8.0)	0.576
Melena	5 (8.3)	47 (6.1)	0.498
Hemoptysis	3 (5.0)	29 (3.8)	0.500
Epistaxis	1 (1.7)	6 (0.8)	0.411
Hematuria	4 (6.7)	71 (9.3)	0.644
**Neurological symptom**	**11 (18.3)**	**285 (37.2)**	0.003*
Apathy	5 (8.3)	159 (20.7)	0.020*
Dysphoria	3 (5.0)	94 (12.3)	0.099
Convulsion	5 (8.3)	119 (15.5)	0.133
Lethargy	2 (3.3)	72 (9.4)	0.156
Confusion	4 (6.7)	147 (19.2)	0.014*
Coma	1 (1.7)	56 (7.3)	0.114
**Plasma leakage**	**9 (15.0)**	**132 (17.2)**	**0.661**
Hydrothorax	8 (13.3)	123 (16.0)	0.581
Hydropericardium	2 (3.3)	16 (2.1)	0.380
Ascites	2 (3.3)	15 (2.0)	0.353
Pelvis fluidify	0 (0.0)	1 (0.1)	1.000
**Respiratory complications**	**37 (61.7)**	**429 (55.9)**	**0.388**
Pulmonary infection	36 (60.0)	368 (48.0)	0.073
Bronchitis	6 (10.0)	176 (23.0)	0.020*
Respiratory failure	0	23 (3.0)	0.402
**Renal failure**	**2 (3.3)**	**20 (2.6)**	**0.670**

Notes: Continuous variables were analysed by *t*-test or nonparametric test; categorical variables were analysed by chi-square test or Fisher’s exact test.

*All *P* values were 2-tailed and *P *<* *.05 was considered statistically significant.

The early symptom of illness was an abrupt onset of fever (98.3%) that began at median of 5 (IQR, 4–7) days before hospital admission. Other frequently seen complaints included malaise (93.3%), myalgia (70.0%), nausea (68.3%), lymphadenopathy (53.3%), cough (50.0%), anorexia (45.0%), productive cough (40.0%), and vomit (30.0%). Other symptoms such as dizziness, arthralgia, and headache were less common. Most of these above mentioned manifestations were presented with a comparable frequency between SFTSV and *R. typhi* infected patients, except that the myalgia was less seen in *R. typhi* infection (70.0% vs. 81.5%, *P *= .030). Only one patient had rash, while four patients had diarrhoea. The diarrhoea was less seen in *R. typhi* than in SFTSV infection (6.7% vs. 22.7%, *P *= .003).

The clinical course was complicated in 42 (70.0%) patients who presented a total of 19 complications, including pulmonary infection in 36 (60.0%), neurological manifestations in 11 (18.3%), and plasma leakage (designates as clinical presence of hydrothorax, hydropericardium or ascites) in 9 (15.0%) of the 60 patients. Both neurological manifestations and presence of bronchitis were significantly lower than that of the SFTSV infection (18.3% vs. 37.2%, *P *= .003; 10.0% vs. 23.0%, *P *= .020; Table 1). Patients who presented with complications were significantly older than those without (age, 60.4 ± 10.3 vs. 53.6 ± 12.3, *P *= .031), and more likely to be male.

Thrombocytopenia and leukopoenia were noted from 41 (68.3%) and 49 (81.7%) cases, respectively (Appendix Table A2). Nine (15.0%) patients displayed anaemia upon admission. The most prominent biochemical finding on admission was the concurrent elevation of aspartate aminotransferase (AST) and lactate dehydrogenase above the normal level, which was observed in 42 (70.0%) patients. Elevated alanine aminotransferase and creatinine were presented in 29 (48.3%) and 32 (53.3%) of the patients, respectively. Hypoalbuminemia was only observed in 5 patients. Altogether 55 (91.7%) patients had abnormal serum electrolyte level, including hyponatremia in 44 (73.3%), hypocalcaemia in 24 (48.0%), hypochloraemia in 9 (15.5%), and hypokalaemia in 32 (53.3%). In comparison with SFTSV infected patients, only thrombocytopenia and elevated AST were significantly underrepresented in *R. typhi* patients (*P *= .001 and *P *= .031, respectively, Appendix Table A2), while others were comparable between two groups. The *R. typhi* infected patients were hospitalized for a median of 10 (IQR, 8–12) days, and fatal outcome was reported from 1 (1.7%) patient (Appendix Tables A3 and A4), significantly lower than those with SFTSV infection (17.1%, *P *< .001). Among the 60 patients, 36 (60.0%) received cephalosporins treatment, 7 (11.7%) received doxycycline treatment, and 6 received levofloxacin treatment. The patients who recovered were followed and no clinical sequelae were observed on one-month observation after discharge from the hospital.

In SFTS endemic region, fever remains one of the major reasons to seek healthcare but the causes remain ill-defined. Here we provided evidence that *R. typhi*, a flea-borne rickettsia was prevalent in this region, with clinical syndromes and laboratory abnormalities highly resembling those with SFTSV infection [10], thus pathogen spectrum of the SFTS should be further expanded. The competent vector of *R. typhi* existed widely in China, especially in regions with warm and humid environment. However, the human cases with *R. typhi* infection were rarely reported and the clinical data were lacking, contributing to a difficulty clinical diagnosis in most of the endemic region in China. Compared to the data from a systematic review [11], several common clinical features of *R. typhi* infection, such as headache, chills, and rash, were less frequently seen in our patients. The main reason for this inconsistency might be that our participants were all recruited from SFTS like patients. We believe that due to the lack of typical signs of *R. typhi* infection, misdiagnosis of *R. typhi* infection was likely to happen in SFTS endemic regions. The elderly patients with *R. typhi* infection were more prone to present severe complications, which was consistent with previous studies [11,14,15]. Due to the possibility of severe complications, the recognition of the *R. typhi* infection and empirical treatment with doxycycline should be stressed, especially for eldly patients in regions with abundant transmission vectors and suitable environment.

The similar clinical manifestations between *R. typhi* and SFTSV infections could be interpreted from the point of pathogenesis that was common between them. *R. typhi* proliferates and spreads via the blood stream causing injury to endothelial and vascular smooth muscle cells, composing the pathophysiologic basis for meningoencephalitis and a skin rash [16]. The haemorrhage might also be caused by increased vascular permeability that is related with the infection. Resembling *R. typhi* infection, pathogenesis of SFTSV infection also involves vascular damage, which leads to an increased vascular permeability and further development of bleeding [17], the damages that underlie the similar complications observed in *R. typhi* infection [16]. The current finding should also be interpreted from the point of coinfection, which might likely happen. According to our previous knowledge, SFTSV coinfection with serological diagnosed spotted fever rickettsia was associated with aggravated disease [10]. Given the potential synergistic effect of SFTSV and *R. typhi* infection, the coinfection should also be warned in SFTS endemic regions.

The study was subject to a major limitation. Due to the fact that event of insect bite or animal contact is most often not recalled by the patients [11], we might miss some of the patients with *R. typhi* infection when using this as one of the inclusion criteria, thus might underestimate the actual infection rate of *R. typhi*. Despite the current limitation, we have confirmed *R. typhi* infection in SFTS like patients, described the clinical and laboratory characteristics of *R. typhi* infection. This information could be helpful for early differentiation between SFTSV and *R. typhi* infection. Further studies are needed to investigate their coinfection in the endemic regions.

## Appendix Appendix

### Molecular test of SFTSV by real-time PCR

Viral RNA was isolated from serum samples using QIAamp Viral RNA Mini Kit (Qiagen), according to the manufacturer’s instructions. The real-time PCR was performed to detect SFTSV RNA with the use of the One step Primer Script RT-PCR Kit (TaKaRa) as previously described [18].

### Molecular test of SFG rickettsia

DNA was isolated from blood specimens collected at admission with the use of the QIAamp Blood Mini Kit (Qiagen) according to manufacturer’s instructions. Nested PCR assays targeting the outer membrane protein A encoding gene (*omp*A), citrate synthase gene (*glt*A), and 17-kDa antigen-encoding gene were concurrently performed to detect the presence of SFG rickettsial DNA. Nucleotide sequences of the primers were shown in Appendix Table A1.

### Molecular test of *Anaplasmataceae*

Nested PCR assays targeting the 16S rRNA gene (Eh16S rRNA) was concurrently performed to detect the presence of *A. phygocytophilum* DNA. Nucleotide sequences of the primers were shown in Appendix Table A1.

### Molecular test of *Babesia*

Nested PCR assays targeting the SSU rRNA gene was concurrently performed to detect the presence of *Babesia microti* DNA. Nucleotide sequences of the primers were shown in Appendix Table A1.

### Molecular test of *B. burgdorferi* sensu lato

The real-time PCR assays targeting the 16S rRNA gene (Bor16SrRNA) was concurrently performed to detect the presence of *B. burgdorferi sensu lato* DNA. Nucleotide sequences of the primers were shown in Appendix Table A1.

Table A1. Nucleotide sequences of primers used in this study.

**Table:**

Gene	Primer	Sequence (5′-3′)	Reference
*omp*A	Rr190.70p	ATGGCGAATATTTCTCCAAAA	[19]
	Rr190.602n	AGTGCAGCATTCGCTCCCCCT	
	190.70-38s1	AAAACCGCTTTATTCACC	[20]
	190.602-384r1	GGCAACAAGTTACCTCCT	
*glt*A	CS1d	ATGACTAATGGCAATAATAA	[21]
	CSEndr	CTTATACTCTCTATGTACA	
	RpCS877p	GGGGACCTGCTCACGGCGG	
	RpCS1258n	ATTGCAAAAAGTACAGTGAACA	
17-kDa	17k-5	GCTTTACAAAATTCTAAAAACCATATA	[22]
	17k-3	TGTCTATCAATTCACAACTTGCC	
	17kD1	GCTCTTGCAACTTCTATGTT	
	17kD2	CATTGTTCGTCAGGTTGGCG	
Eh16S rRNA	Eh-out1	TTGAGAGTTTGATCCTGGCTCAGAACG	[23]
	Eh-out2	CACCTCTACACTAGGAATTCCGCTATC	
	Eh-gs1	GTAATAACTGTATAATCCCTG	
	Eh-gs2	GTACCGTCATTATCTTCCCTA	
SSU rRNA	Bab1	CTTAGTATAAGCTTTTATACAGC	[24]
	Bab4	ATAGGTCAGAAACTTGAATGATACA	
	Bab2	GTTATAGTTTATTTGATGTTCGTTT	
	Bab3	AAGCCATGCGATTCGCTAAT	
Bor16SrRNA	BorITS4F	GGCTTCGGGTCTACCACATCTA	[25]
	BorITS4R	CCGGGAGGGGAGTGAAATAG	
	BorITS4PFAM	FAMTGCAAAAGGCACGCCATCACCBHQ1	

Table A2. Laboratory test results of the patients with *R. typhi* infection vs. SFTSV infection, 2014–2015.

**Table:**

Laboratory parameters	R. typhi patients (n = 60)	SFTSV patients (n = 767)	P-value
WBC count < 4 × 109/L	49 (81.7)	632 (82.4)	0.886
PLT count < 100 × 109/L	41 (68.3)	648 (84.5)	0.001*
Neutrophils > 70%	30 (50.0)	350 (45.6)	0.513
Lymphocytes < 20%	24 (40.0)	278 (36.3)	0.561
HGB < 110 g/L	9 (15.0)	114 (14.9)	0.977
AST > 40 U/L	44 (73.3)	645 (84.1)	0.031*
ALT > 40 U/L	29 (48.3)	438 (57.1)	0.187
ALB < 35 g/L	5 (8.3)	95 (12.4)	0.354
ALP > 150 U/L	2 (3.3)	49 (6.4)	0.574
GGT > 50 U/L	13 (21.7)	174 (22.7)	0.856
LDH > 245 U/L	48 (80.0)	654 (85.3)	0.273
CK > 200 U/L	32 (53.3)	482 (62.8)	0.144
BUN > 7.14 mmol/L	19 (31.7)	247 (32.3)	0.921
CREA > 97 μmol/L	6 (10.0)	147 (19.2)	0.077
AMY > 115 U/L	11 (26.8)	190 (39.9)	0.099
Ca < 2.1 mmol/L	24 (48.0)	399 (59.4)	0.115
Cl < 95 mmol/L	9 (15.5)	89 (12.0)	0.426
K < 3.5 mmol/L	32 (53.3)	331 (43.2)	0.128
Na < 135 mmol/L	44 (73.3)	530 (69.2)	0.502

Notes: All *P* values were 2-tailed and *P *<* *.05 was considered statistically significant. Categorical variables were analysed by chi-square test or Fisher’s exact test. WBC: White blood cell; PLT: Platelet count; HGB: Haemoglobin; AST: Aspartate aminotransferase; ALT: Alanine transaminase; ALB: Albumin; ALP: Alkaline phosphatase; GGT: γ-glutamyl transpeptadase; LDH: Lactate dehydrogenase; CK: Creatine kinase; BUN: Blood urea nitrogen; CREA: Creatinine; AMY: Serum amylase; Ca: Calcium; Cl: Chlorine; K: Potassium; Na: Natrium.

Table A3. Demographic and clinical characteristics of the patient with fatal *R. typhi* infection.

**Table:**

Characteristics	
*Demographic characteristics*	
Age, years	64
Sex	Male
Reported history of insect bite	−
Hospital duration	8
Delay days from onset to admission	4
Underlying diseases	Hypertension
*Clinical manifestations*	
*Common signs*	
Fever	+
Maximum temperature	40
Chills	−
Malaise	+
Lymphadenectasis	+
Myalgia	+
Dizziness	−
Headache	−
Rash	−
Arthralgia	−
*Respiratory syndrome*	
Cough	+
Productive cough	−
*Gastrointestinal illness*	
Anorexia	+
Nausea	+
Vomit	+
Abdominal pain	−
Abdominal distension	−
Diarrhoea	−
*Haemorrhage manifestations*	
Gingival bleeding	−
Melena	+
Hemoptysis	−
Epistaxis	−
Hematuria	−
Ecchymosis	+
*Neurological symptom*	
Apathy	+
Dysphoria	+
Convulsion	+
Lethargy	+
Confusion	+
Coma	+
*Plasma leakage*	
Hydrothorax	−
Hydropericardium	−
Ascites	−
Pelvis fluidify	−
*Respiratory complications*	
Pulmonary infection	+
Bronchitis	+
Respiratory failure	+

Table A4. Laboratory test results of the patient with fatal *R. typhi* infection.

**Table:**

Laboratory parameters	Normal range	Patient
WBC count (×10^9^/L)	4.0–10.0	5.0
PLT count (×10^9^/L)	100–300	57
Neutrophils (%)	50.0–70.0	83.6
Lymphocytes (%)	20.0–40.0	14.0
HGB (g/L)	110–170	136
AST (U/L)	0–40	51
ALT (U/L)	0–40	29
ALB (g/L)	35.0–55.0	39.3
ALP (U/L)	40–150	85
GGT (U/L)	7–50	15
LDH (U/L)	109–245	313
CK (U/L)	25–200	92
BUN (mmol/L)	1.4–7.1	8.94
CREA (μmol/L)	40–97	98
AMY (U/L)	25–115	129
Ca (mmol/L)	2.1–2.7	2.12
Cl (mmol/L)	95–108	101
K (mmol/L)	3.5–5.5	3.6
Na (mmol/L)	135–155	131

Notes: WBC: White blood cell; PLT: Platelet count; HGB: Haemoglobin; AST: Aspartate aminotransferase; ALT: Alanine transaminase; ALB: Albumin; ALP: Alkaline phosphatase; GGT: γ-glutamyl transpeptadase; LDH: Lactate dehydrogenase; CK: Creatine kinase; BUN: Blood urea nitrogen; CREA: Creatinine; AMY: Serum amylase; Ca: Calcium; Cl: Chlorine; K: Potassium; Na: Natrium.
